# Supplementary material for: lagCI enables inference of temporal causal relationships from dense multi-omic time series
Source: Brief Bioinform. 2026 Sep 9;27(5):bbag464. doi: 10.1093/bib/bbag464 (PMC13557713; doi:10.1093/bib/bbag464)
Supplement: Supplementary file 1 [file lagci_final_supplementary_file_bbag464.docx]

**Supplementary Information for**

**LagCI Enables Inference of Temporal Causal Relationships From Dense Multi-Omic Time Series**

Yifei Ge^1,2,+^, Shunpeng Bai^1,3,+^, Zirui Qiang^1,4,+^, Yijiang Liu^1,2,+^, Yitong Wu^5^, Xiaotao Shen^1,2,6,*^

1. Lee Kong Chian School of Medicine, Nanyang Technological University, Singapore, Singapore
2. School of Chemistry, Chemical Engineering and Biotechnology, Nanyang Technological University, Singapore, Singapore
3. College of Life Science and Technology, Huazhong Agricultural University, Wuhan, Hubei 430070, China
4. College of Computer Science and Technology, Harbin Institute of Technology, Shenzhen, Guangdong 518055, China
5. School of Biological Sciences, The University of Hong Kong, Hong Kong, China
6. Singapore Phenome Center, Lee Kong Chian School of Medicine, Nanyang Technological University, Singapore, Singapore

^+^ These authors contributed equally: Yifei Ge, Shunpeng Bai, Zirui Qiang, and Yijiang Liu.

^*^ Corresponding authors: Xiaotao Shen ([xiaotao.shen@ntu.edu.sg](mailto:xiaotao.shen@ntu.edu.sg)).

# Supplementary Figure

## Supplementary Figure 1. Hyperparameter and threshold sensitivity analyses of lagCI. (a) Receiver operating characteristic (ROC) analysis showing the ability of absolute correlation and quality score to distinguish real simulated temporal relationships from permutation-null pairs. (b) Direction recovery and fraction of retained participants across absolute-correlation and quality-score thresholds. (c) Direction recovery of the expected steps-to-heart-rate relationship across correlation and interpolation configurations in the smartwatch validation dataset. (d) Median absolute correlation and quality score for each configuration.

## Supplementary Figure 2. Temporal-resolution analysis and benchmarking on smartwatch data. (a) lagCI direction recovery after subsampling the smartwatch data to different temporal resolutions. (b) Direction-recovery comparison of lagCI, Granger causality, and PCMCI for the known steps → heart rate relationship. Strict requires only the correct direction to be significant; dominant selects the direction with the lower p value.

## Supplementary Figure 3. Distribution of multi-omics measurements across the sampling period.

# Supplementary Note

## lagCI software

*Software implementation.* We developed lagci, an R package for time-resolved association analysis between two longitudinal signals, particularly for wearable and omics data. The package was implemented in R (version ≥ 4.1) and distributed as an MIT-licensed package. Core imports include stats, lubridate, BiocParallel, ggplot2, dplyr, massdataset, and related visualization and utility packages, enabling time handling, interpolation, correlation analysis, parallel computation, and result visualization within a unified workflow

The package exposes a compact set of primary analysis functions, including calculate_lagged_correlation(), smooth_data(), fit_smoothing_parameters(), and evaluate_lagged_cor(), together with accessor and plotting functions for downstream interpretation of lagged correlation profiles and aligned time-series relationships.

*Data model and result object.* Lagged correlation results are stored in an S4 class, lagged_cor_result, which retains the original input vectors (x, y), their timestamps (time1, time2), the full lag-resolved correlation profile (all_cor), corresponding p values (all_cor_p), lag interval labels (shift_time), the index of the maximum absolute correlation (which_max_idx), the index corresponding to the zero-lag correlation (which_global_idx), the maximum correlation value (max_cor), the zero-lag correlation value (global_cor), and a structured parameter record (tidymass_parameter) describing the analysis settings used to generate the result. This object design allows the full lagged-correlation landscape, rather than only a single summary statistic, to be preserved for downstream scoring and visualization.

*Optional preprocessing and smoothing of time-series data.* To reduce high-frequency noise prior to downstream analysis, lagci implements an optional LOESS-based smoothing workflow. In smooth_data(), the observed signal is modeled as a function of numeric time using loess(x ~ as.numeric(time)), with user-specified span and polynomial degree, and the fitted values are returned together with the original data. An optional plotting mode overlays the original and smoothed trajectories for visual inspection of smoothing behavior.

To assist parameter selection, the package also provides fit_smoothing_parameters(), which performs a grid search over candidate LOESS span and degree values. The input series is first sorted by time, and the first one-third of the observations is used as a fitting subset. After excluding missing values and values ≤ 0, LOESS models are fit across the candidate parameter grid in parallel using BiocParallel. Each model is then used to predict the full original series, and the squared Pearson correlation (R²) between observed and predicted values is calculated. The final smoothing parameters are chosen as the combination whose R² is closest to 0.6, rather than the absolute maximum R², thereby favoring moderate smoothing over potentially overfit trajectories. By default, span values from 0.1 to 0.5 in increments of 0.01 and polynomial degrees 1 or 2 are evaluated.

*Calculation of lagged correlation.* The main analysis function, calculate_lagged_correlation(), estimates the correlation between two time-series signals across a user-defined lag window. Inputs include two numeric vectors (x and y), their timestamp vectors (time1 and time2), the total lag tolerance (time_tol, in hours), the lag step size (step, in hours), the minimum number of matched observations required for a valid analysis, the alignment method, and the correlation method (spearman or pearson).

Before alignment, both input vectors are converted to numeric and standardized using z-score scaling. The analysis is restricted to the overlapping time interval defined by the later of the two start times and the earlier of the two end times. A regular time grid is then generated across this shared interval at the requested temporal resolution (step × 60 minutes). Each input series is interpolated onto this common grid using stats::approx(), with either linear or constant interpolation and with edge extrapolation enabled (rule = 2) to avoid missing values at the boundaries of the overlap interval

For Spearman analysis, the aligned values are rank-transformed prior to correlation calculation; for Pearson analysis, the aligned values are used directly. Complete paired observations are retained, and analyses are discarded if the number of effective matched samples is smaller than either 2 or the user-specified min_matched_sample threshold. The maximum lag index is computed as round(time_tol / step) - 1 and capped at n_eff - 1, where n_eff is the number of complete aligned observations. Lagged correlations are then calculated using the discrete cross-correlation function (stats::ccf) without plotting.

From the ccf output, the package extracts the full lag-dependent correlation profile (all_cor) and computes two-sided p values by normal approximation under a null centered at zero, using a standard deviation of 1 / sqrt(n_used), where n_used is the number of observations used in the cross-correlation calculation. The lag axis is represented as consecutive shift intervals centered on the integer lag positions and expressed in minutes. The package records both the lag with the maximum absolute correlation and the zero-lag position, allowing users to distinguish delayed associations from synchronous associations within the same result object.

*Evaluation of lagged-correlation profile shape.* Beyond identifying the largest lagged association, lagci implements an additional profile-level scoring procedure in evaluate_lagged_cor(). This function takes a lagged_cor_result object and evaluates whether the lag-correlation curve exhibits a coherent peak-like structure rather than a noisy or fragmented profile. To do this, the function first converts the lag-interval labels into numeric shift times by taking the midpoint of each lag interval. Missing correlation values are set to zero, and a LOESS curve is fit to the lag-correlation profile as a function of shift time. The smoothing span is set adaptively to 3 / length(cor) with a lower bound of 0.2, and predictions are generated on a 1-minute grid spanning the lag range.

The smoothed lag-correlation curve is then decomposed into positive and negative candidate envelopes. For the positive component, the curve is shifted upward if needed so that all values are non-negative, the dominant positive peak is identified, and a Gaussian peak shape is reconstructed using fitted location, width, and area parameters. The similarity between the reconstructed peak and the positive envelope is quantified using Spearman correlation. The same procedure is repeated for the negative component after sign inversion. The final score is defined as the larger of the positive and negative peak-fit scores, provided that the original lag-correlation profile actually contains a positive or negative extremum of the corresponding sign. When plotting is enabled, the function returns a visualization showing the smoothed lag-correlation profile, the fitted peak, the significance of observed lag bins, and the zero-shift reference line.

*Visualization and interpretation of lagged associations.* The package includes dedicated plotting utilities to visualize lagged relationships from complementary perspectives. lagged_alignment_plot() displays the temporal alignment between the two input series at either the zero-lag (“global”) position or the lag corresponding to the maximum absolute correlation, with options to display matched points, integrated averages, and connection lines between aligned observations. lagged_scatter_plot() summarizes the relationship between the two signals at a selected lag by plotting matched values as either a scatter plot or a hexbin plot and overlays a fitted linear trend for visual interpretation of the lag-specific association strength.

*Parallelization and reproducibility.* Where applicable, computationally intensive steps are parallelized using BiocParallel. In fit_smoothing_parameters(), parallel backends are chosen in an operating-system-aware manner: SnowParam is used on Windows and MulticoreParam on non-Windows systems. A random seed can be supplied to ensure reproducible parameter selection across runs. In calculate_lagged_correlation(), analysis parameters are recorded in a tidymass_parameter object and stored in the returned lagged_cor_result, preserving the exact settings used for lag tolerance, step size, sample threshold, thread count, and correlation method.

**Permutation-null analysis of lagCI filtering scores**

To assess whether lagCI-derived scores distinguish true temporal structure from noise, we generated permutation-null pairs from the simulated datasets by randomly shuffling one time series in each pair. lagCI was applied to both original and permuted pairs using the same settings as the simulation benchmark. The quality score and absolute maximum lagged correlation were extracted for each pair, and their discriminative ability was evaluated by receiver operating characteristic (ROC) analysis, with original simulated pairs treated as positives and permuted pairs as negatives. Performance was summarized by the area under the ROC curve (AUC).

**Top-k recovery analysis**

To evaluate whether the top-ranked lagCI results were enriched for true temporal relationships, we performed a top-k recovery analysis using the simulated benchmark datasets. For each simulated dataset, lagCI-predicted pairs were ranked by the absolute maximum lagged correlation. Each predicted pair was then compared with the predefined ground-truth causal edges used to generate the simulation. A pair was counted as true if it matched a known causal relationship in either direction. We then calculated how many true relationships were recovered among the top 10, top 20, and top 50 ranked pairs. Ranking performance was further summarized using precision@k and average precision, which measures how consistently true relationships appear near the top of the ranked list.

## Hyperparameter and threshold sensitivity analysis

We assessed the robustness of lagCI using the smartwatch validation dataset, where the expected causal direction is from step count to heart rate. We compared four parameter combinations, including Spearman or Pearson correlation with linear or constant interpolation, while keeping other settings consistent with the main analysis. Directional recovery was defined as the fraction of participants in which lagCI recovered the expected steps-to-heart-rate direction.

Threshold sensitivity was evaluated by varying the quality-score and absolute maximum lagged-correlation cutoffs and recording both direction recovery and the fraction of retained pairs. In addition, simulated causal time-series pairs were compared with matched permutation-null pairs, generated by shuffling one time series, to assess whether these scores could distinguish true temporal relationships from noise using receiver operating characteristic analysis.

## Temporal-resolution analysis on smartwatch data

To evaluate the effect of temporal resolution on lagCI performance, we used the smartwatch dataset. Starting from the original 1-min step-count and heart-rate time series, we generated subsampled datasets at 2, 5, 10, 15, 30, and 60 min resolution for each participant. At each resolution, lagCI was re-applied using the same 30-min maximum lag window. Direction recovery was defined as successful when the inferred lag indicated that step activity preceded heart-rate response. Cases in which the peak lag collapsed to zero were treated as ambiguous rather than incorrect.

## Benchmarking on smartwatch data

To compare lagCI with established temporal causal inference methods, we benchmarked lagCI against Granger causality and PCMCI implemented in Tigramite using the smartwatch dataset. Time series were aligned on a 1-min grid, and participants with sufficient overlapping measurements were retained for analysis. The expected ground-truth direction was defined as steps → heart rate. For Granger causality and PCMCI, both steps → heart rate and heart rate → steps were tested within a 30-min maximum lag window using a nominal significance threshold of p value < 0.05. Direction recovery was summarized using two criteria: a strict criterion requiring only the correct direction to be significant, and a dominant-direction criterion in which the direction with the lower p value was selected when both directions were significant.

## Computational scalability analysis

To evaluate the computational scalability of lagCI, we analyzed both the theoretical per-pair complexity and empirical runtime and memory scaling. For each pairwise lagCI calculation, we denoted the time-series length as n and the number of tested lag positions as L. The per-pair computational cost consists of time-series alignment/interpolation and lagged-correlation scanning across L lag positions, giving a theoretical time complexity of O(n log n + L·n) and a space complexity of O(n + L). Since each molecular pair is evaluated independently, large-scale pairwise analyses are naturally parallelizable across pairs.

Empirical scalability was assessed using controlled single-pair benchmarks. We varied the time-series length n while keeping the lag-search setting fixed, and separately varied the number of tested lag positions L while keeping n fixed. All benchmark runs were performed in single-thread mode to estimate the baseline per-pair computational cost. Runtime and memory usage were recorded for each setting, and empirical scaling exponents were estimated by fitting log-log linear models between input size and runtime or memory usage. The resulting runtime and memory scaling summaries are reported in Supplementary Table 3.

## Reproducible lagCI workflow using the built-in demonstration dataset

To demonstrate how to reproduce a simple lagged-correlation inference using lagCI, we provide a short example based on the built-in demonstration dataset included in the lagCI/lagcishiny workflow. The example contains two time-series variables: step count and heart rate. This toy dataset was used to illustrate a known temporal relationship in which changes in physical activity precede changes in heart rate.

In this example, we set step count as x and heart rate as y. The shift time is defined as x - y. Therefore, a negative shift time indicates that changes in step count occur before changes in heart rate, whereas a positive shift time indicates that changes in step count occur after changes in heart rate.

1. **Load packages and demonstration data**

library(lagci)

library(ggplot2)

\# Load the built-in demonstration datasets.

\# Depending on the installed package version, these data can be loaded

\# directly from lagCI or from the associated demo-data package.

data("heart_data")

data("step_data")

head(heart_data, 5)

head(step_data, 5)

Example output:

```
##                  time heart
## 1 2019-04-29 00:00:08    64
## 2 2019-04-29 00:00:13    65
## 3 2019-04-29 00:00:18    66
## 4 2019-04-29 00:00:23    65
## 5 2019-04-29 00:00:33    64
##                  time step
## 1 2019-04-29 00:00:00    0
## 2 2019-04-29 00:01:00    0
## 3 2019-04-29 00:02:00    0
## 4 2019-04-29 00:03:00    0
## 5 2019-04-29 00:04:00    0
```

2. **Visualize the two input time series**

p_heart <- lagci::time_plot(

x = heart_data$heart,

time = heart_data$time,

y_axis_name = "Heart rate"

)

p_step <- lagci::time_plot(

x = step_data$step,

time = step_data$time,

y_axis_name = "Step count"

)

p_heart #Time-series plot of heart rate.

p_step #Time-series plot of step count

3. **Run lagCI**

We next applied lagCI to calculate lagged correlations between step count and heart rate. Step count was used as x, and heart rate was used as y.

x <- step_data$step

time1 <- step_data$time

y <- heart_data$heart

time2 <- heart_data$time

result <- lagci::calculate_lagged_correlation(

x = x,

y = y,

time1 = time1,

time2 = time2,

time_tol = 0.2,

step = 2 / 60,

min_matched_sample = 10,

progressbar = TRUE,

threads = 10,

cor_method = "spearman"

)

The key parameters used in this example were:

```
## time_tol: 0.2
## step: 0.0333333333333333
## min_matched_sample: 10
## progressbar: TRUE
## threads: 10
## cor_method: spearman
```

The input data contained 12,960 observations for step count and 91,948 observations for heart rate:

```
## length of x: 12960
## length of y: 91948
```

4. **Extract lagCI output**

The maximum lagged correlation was extracted using **extract_max_cor()**:

lagci**::extract_max_cor(result)**

Output:

```
## (-3,-1]
## 0.59137
```

The global, near-zero-lag correlation was extracted using **extract_global_cor()**:

lagci**::extract_global_cor(result)**

Output:

```
## (-1,1]
## 0.5336532
```

The tested shift-time windows were extracted using **extract_shift_time()**:

list(

numeric = lagci::extract_shift_time(result, numeric = TRUE),

annotated = lagci::extract_shift_time(result, numeric = FALSE)

)

Output:

```
## $numeric
## [1] -10  -8  -6  -4  -2   0   2   4   6   8  10
##
## $annotated
## [1] "(-11,-9]" "(-9,-7]"  "(-7,-5]"  "(-5,-3]"  "(-3,-1]"  "(-1,1]"
## [7] "(1,3]"    "(3,5]"    "(5,7]"    "(7,9]"    "(9,11]"
```

The quality score of the lagged-correlation profile was calculated using **evaluate_lagged_cor()**:

suppressWarnings(

lagci::evaluate_lagged_cor(result, plot = FALSE)$score

)

Output:

\## [1] 0.9869825

5. **Visualize the lagged-correlation profile and aligned signals**

The lagged-correlation profile was visualized using the evaluation function:

lag_profile <- lagci::evaluate_lagged_cor(

result,

plot = TRUE

)

lag_profile$plot #Evaluated lagged-correlation profile across tested shift-time windows

The maximum-correlation alignment plot was generated as follows:

p_align_max <- lagci::lagged_alignment_plot(object = result,

x_color = "blue",

y_color = "red",

x_name = "x",

y_name = "y",

which = "max",

x_limit = c(1,100000),

non_matched_point_size = 0.1,

y_point_size = 1,

x_point_size = 1,

integrated = FALSE,

add_connect_line = FALSE,

add_point = TRUE,

time_gap = 12)

p_align_max #Alignment plot at the maximum-correlation shift window.

The corresponding scatter plot was generated as follows:

p_scatter_max <- lagci::lagged_scatter_plot(

object = result,

x_name = "Step count",

y_name = "Heart rate",

which = "max",

hex = TRUE

)

p_scatter_max # Hexbin scatterplot at the maximum-correlation shift window.

## R packages used in the study

R version 4.5.2 (2025-10-31)

Platform: aarch64-apple-darwin20

Running under: macOS Tahoe 26.3.1

Matrix products: default

BLAS: /System/Library/Frameworks/Accelerate.framework/Versions/A/Frameworks/vecLib.framework/Versions/A/libBLAS.dylib

LAPACK: /Library/Frameworks/R.framework/Versions/4.5-arm64/Resources/lib/libRlapack.dylib; LAPACK version 3.12.1

locale:

[1] en_US.UTF-8/en_US.UTF-8/en_US.UTF-8/C/en_US.UTF-8/en_US.UTF-8

time zone: Asia/Singapore

tzcode source: internal

attached base packages:

[1] tcltk	stats	graphics grDevices utils	datasets methods	base

other attached packages:

[1] vegan_2.7-1	permute_0.9-8	scales_1.4.0	pheatmap_1.0.13	Mfuzz_2.68.0	DynDoc_1.86.0

[7] widgetTools_1.86.0 e1071_1.7-16	Biobase_2.68.0	BiocGenerics_0.54.0 generics_0.1.4	lubridate_1.9.4

[13] forcats_1.0.0	stringr_1.5.1	dplyr_1.1.4	purrr_1.1.0	readr_2.1.5	tidyr_1.3.1

[19] tibble_3.3.0	ggplot2_4.0.2	tidyverse_2.0.0	magrittr_2.0.3	r4projects_0.1.7

loaded via a namespace (and not attached):

[1] DBI_1.2.3	tmaptools_3.3	remotes_2.5.0	rlang_1.1.6

[5] ade4_1.7-23	clue_0.3-66	GetoptLong_1.0.5	matrixStats_1.5.0

[9] compiler_4.5.2	mgcv_1.9-3	reshape2_1.4.4	png_0.1-8

[13] systemfonts_1.2.3	vctrs_0.6.5	rvest_1.0.4	pkgconfig_2.0.3

[17] shape_1.4.6.1	crayon_1.5.3	XVector_0.48.0	lwgeom_0.2-14

[21] labeling_0.4.3	utf8_1.2.6	tzdb_0.5.0	UCSC.utils_1.4.0

[25] ragg_1.4.0	bit_4.6.0	GenomeInfoDb_1.44.2	jsonlite_2.0.0

[29] biomformat_1.36.0	rhdf5filters_1.20.0	DelayedArray_0.34.1	Rhdf5lib_1.30.0

[33] parallel_4.5.2	cluster_2.1.8.1	R6_2.6.1	stringi_1.8.7

[37] RColorBrewer_1.1-3	GenomicRanges_1.60.0	stars_0.6-8	Rcpp_1.1.0

[41] SummarizedExperiment_1.38.1 iterators_1.0.14	IRanges_2.42.0	igraph_2.1.4

[45] splines_4.5.2	rentrez_1.2.4	Matrix_1.7-4	timechange_0.3.0

[49] tidyselect_1.2.1	rstudioapi_0.17.1	stringdist_0.9.15	dichromat_2.0-0.1

[53] abind_1.4-8	doParallel_1.0.17	codetools_0.2-20	plyr_1.8.9

[57] lattice_0.22-7	withr_3.0.2	S7_0.2.0	survival_3.8-3

[61] sf_1.0-21	units_0.8-7	proxy_0.4-27	zip_2.3.3

[65] xml2_1.4.0	Biostrings_2.76.0	circlize_0.4.16	phyloseq_1.52.0

[69] pillar_1.11.0	MatrixGenerics_1.20.0	tkWidgets_1.86.0	KernSmooth_2.23-26

[73] foreach_1.5.2	stats4_4.5.2	vroom_1.6.5	S4Vectors_0.48.0

[77] hms_1.1.3	class_7.3-23	glue_1.8.0	tools_4.5.2

[81] data.table_1.17.8	ggsignif_0.6.4	openxlsx_4.2.8	XML_3.99-0.19

[85] rhdf5_2.52.1	grid_4.5.2	ape_5.8-1	massdataset_0.99.3

[89] colorspace_2.1-1	nlme_3.1-168	GenomeInfoDbData_1.2.14	cli_3.6.5

[93] textshaping_1.0.1	S4Arrays_1.8.1	ComplexHeatmap_2.24.1	gtable_0.3.6

[97] digest_0.6.37	classInt_0.4-11	SparseArray_1.8.1	rjson_0.2.23

[101] farver_2.1.2	multtest_2.64.0	lifecycle_1.0.4	httr_1.4.7

[105] GlobalOptions_0.1.2	MASS_7.3-65	bit64_4.6.0-1

# Supplementary Tables

### Supplementary Table 1. Wearable individual-level lagCI results

| id | lag_center_min | max_cor | global_cor | quality_score |
| --- | --- | --- | --- | --- |
| A06L7KF | -1 | 0.514741987 | 0.502253149 | 0.979544126 |
| A0822M0 | -2 | 0.582396984 | 0.485506655 | 0.972472238 |
| A0KX894 | -2 | 0.398097241 | 0.347151115 | 0.947340736 |
| A0L9BM2 | -1 | 0.485373215 | 0.447154144 | 0.984862653 |
| A0N9NV4 | -1 | 0.472216692 | 0.449269941 | 0.949444769 |
| A0NVTRV | -2 | 0.121059266 | 0.105145817 | 0.9471654 |
| A0VFT1N | -1 | 0.460630254 | 0.434315022 | 0.985797779 |
| A11SQQN | -1 | 0.344435002 | 0.308962928 | 0.966510812 |
| A11V1FH | -2 | 0.498078735 | 0.455573362 | 0.972998247 |
| A17YCA2 | -2 | 0.577368195 | 0.52811414 | 0.981297487 |
| A1K5DRI | -1 | 0.553551696 | 0.536278653 | 0.978667446 |
| A1ZJ41O | -2 | 0.448312398 | 0.387009771 | 0.966101695 |
| A2D7K4A | -2 | 0.413277332 | 0.361124323 | 0.981180596 |
| A2P3LTM | -2 | 0.503615637 | 0.469837532 | 0.976271186 |
| A2XFW2N | -1 | 0.067810854 | 0.054861135 | 0.87182934 |
| A35BJNV | -2 | 0.422317702 | 0.372048175 | 0.987960257 |
| A36HR6Y | -1 | 0.245567034 | 0.216737504 | 0.959964933 |
| A3OU183 | -1 | 0.272910194 | 0.263743062 | 0.981940386 |
| A45F9E6 | -2 | 0.343137024 | 0.273749916 | 0.974284044 |
| A4E0D03 | -2 | 0.509205817 | 0.436776998 | 0.966569258 |
| A4G0044 | -1 | 0.485523242 | 0.464415049 | 0.957276447 |
| A4H7SNF | -2 | 0.534571545 | 0.471155674 | 0.989830508 |
| A5XL2IC | -1 | 0.584941477 | 0.471022652 | 0.980713033 |
| A65HVGP | -1 | 0.595351402 | 0.554042531 | 0.998129749 |
| A6BUI4N | -2 | 0.637700792 | 0.577742306 | 0.990414962 |
| A6GEBIK | -2 | 0.428687275 | 0.348037262 | 0.977206312 |
| A7EAWA7 | -2 | 0.446354098 | 0.39766012 | 0.97936879 |
| A7EM0B6 | -2 | 0.502214271 | 0.411399496 | 0.983167738 |
| A8CBEJZ | -2 | 0.625281459 | 0.567389093 | 0.986732905 |
| A8QLAB0 | -1 | 0.437231937 | 0.417501115 | 0.959848042 |
| A91HEZV | -1 | 0.63978545 | 0.599289847 | 0.9735827 |
| A99ZKKW | -2 | 0.321356973 | 0.304567968 | 0.978550555 |
| A9ZG5GR | -2 | 0.521617278 | 0.480833112 | 0.952308591 |
| AA0HAI1_1 | -1 | 0.253945985 | 0.206796394 | 0.975803624 |
| AA0HAI1_2 | -1 | 0.271152393 | 0.187982306 | 0.960081823 |
| AA0HAI1_3 | -2 | 0.252170332 | 0.15481091 | 0.952542373 |
| AA2KP1S | -2 | 0.473157719 | 0.394364625 | 0.977673875 |
| AAF9ACE | -2 | 0.557013464 | 0.503441284 | 0.973056692 |
| AAGTWZK | -1 | 0.546770191 | 0.519309227 | 0.960900058 |
| AAXAA7Z | -2 | 0.595243042 | 0.524391983 | 0.986732905 |
| AD77K91 | -1 | 0.340243735 | 0.330220448 | 0.961075395 |
| AE0MQ94 | -1 | 0.589414946 | 0.576956914 | 0.992986558 |
| AE2B3RH | -1 | 0.359406936 | 0.327185931 | 0.969725307 |
| AEOBCFJ | -2 | 0.612754739 | 0.569373257 | 0.988077148 |
| AEOHH30 | -2 | 0.471835666 | 0.401614912 | 0.971186441 |
| AEZDKVO | -2 | 0.364366859 | 0.287348711 | 0.988603156 |
| AF3J1YC | -3 | 0.564116438 | 0.511816478 | 0.980420807 |
| AF3MXM1 | -2 | 0.559361233 | 0.503744474 | 0.963588545 |
| AF8R0I6 | -1 | 0.542696122 | 0.504583631 | 0.979485681 |
| AFEFA29 | -1 | 0.408998262 | 0.375315335 | 0.975920514 |
| AFHOHOM | -2 | 0.281518701 | 0.2450562 | 0.974810053 |
| AFPB8J2 | -1 | 0.522273149 | 0.456600149 | 0.950730567 |
| AFVAEC7 | -1 | 0.463728939 | 0.446414631 | 0.980362361 |
| AFYLHG4 | -1 | 0.312304636 | 0.293674424 | 0.967036821 |
| AGA8XUN | -1 | 0.458086797 | 0.444793987 | 0.990648743 |
| AGKI03N | -2 | 0.315039461 | 0.269500814 | 0.994389246 |
| AGYQJEL | -2 | 0.660037039 | 0.616228807 | 0.987258913 |
| AHP25OJ | -1 | 0.286551221 | 0.255221751 | 0.955289305 |
| AHYIJDV | -2 | 0.419180317 | 0.375273526 | 0.982057276 |
| AHYR55C | -1 | 0.177506006 | 0.176803682 | 0.921040327 |
| AIFDJZB | -2 | 0.604119303 | 0.538629635 | 0.98351841 |
| AJ0DKQ3 | -2 | 0.622993018 | 0.575533924 | 0.978082992 |
| AJ7TSV9 | -2 | 0.325017833 | 0.275101307 | 0.976154296 |
| AJMQUVV | -2 | 0.499427175 | 0.46052824 | 0.974926943 |
| AJWW3IY | -1 | 0.456155678 | 0.41763402 | 0.973641146 |
| AK7YRBU | -2 | 0.334108878 | 0.275564989 | 0.99333723 |
| AKTGD8X | -1 | 0.527568937 | 0.515584125 | 0.989596727 |
| AKV66US | -2 | 0.277142019 | 0.261009731 | 0.960724722 |
| AKXN5ZZ | -2 | 0.49183285 | 0.409731073 | 0.964640561 |
| AL3KT5B | -2 | 0.629191157 | 0.581068455 | 0.954412624 |
| AL48GP3 | -2 | 0.620901425 | 0.558663961 | 0.979485681 |
| ALKAXMZ | -1 | 0.517171388 | 0.495257143 | 0.977556984 |
| ALZDAVZ | -2 | 0.462608727 | 0.401658088 | 0.990414962 |
| AMQUHOQ | -1 | 0.448833835 | 0.434106368 | 0.992109877 |
| AMV7EQF | -2 | 0.414301078 | 0.39226991 | 0.994038574 |
| AO20DS4 | -2 | 0.423526964 | 0.366200867 | 0.958679135 |
| AOB9SON | -2 | 0.53561187 | 0.510901856 | 0.984862653 |
| AOGFRXL | -2 | 0.682223452 | 0.601570469 | 0.981239041 |
| AOQA85X | -1 | 0.518782635 | 0.503499699 | 0.985388662 |
| AOS4BSJ | -1 | 0.560494609 | 0.541989125 | 0.990005845 |
| AOYM4KG | -1 | 0.523342934 | 0.475957541 | 0.974868498 |
| APDJ1QP | -3 | 0.558216064 | 0.518348881 | 0.973641146 |
| APGIB2T | -1 | 0.469264785 | 0.454805838 | 0.984102864 |
| APHNRSV | -1 | 0.260520512 | 0.199534591 | 0.983635301 |
| AQ25Y0L | -2 | 0.463993755 | 0.394957418 | 0.974400935 |
| AQ4TMLV | -2 | 0.519334185 | 0.486000295 | 0.975160725 |
| AQC0L71 | -1 | 0.533341467 | 0.500885766 | 0.985856224 |
| AQR8ZSS | -1 | 0.530889193 | 0.525884183 | 0.989713618 |
| AR4FPCC | -1 | 0.627674859 | 0.595074311 | 0.987784921 |
| ARFYLMK | -2 | 0.504756888 | 0.462558628 | 0.988836937 |
| ARR2IKE | -2 | 0.366838144 | 0.325690486 | 0.952600818 |
| ARYB2QO | -2 | 0.54296683 | 0.507632339 | 0.988135593 |
| AS2MVDL | -1 | 0.334167625 | 0.328039705 | 0.9421391 |
| ASFODQR | -2 | 0.568365176 | 0.536025995 | 0.975686733 |
| ATHBFWX | -2 | 0.51051866 | 0.464274324 | 0.972355348 |
| ATHKM6V | -2 | 0.164923601 | 0.083839621 | 0.940268849 |
| ATT9RR1 | -1 | 0.465272757 | 0.420144721 | 0.963062537 |
| AUCGUF3 | -2 | 0.562297842 | 0.490017124 | 0.946230275 |
| AUILKHG | -1 | 0.432987242 | 0.404546458 | 0.947574518 |
| AURCTAK | -1 | 0.482616562 | 0.468609646 | 0.979310345 |
| AUY8KYW | -1 | 0.646110521 | 0.590229301 | 0.99421391 |
| AV2GF3B | -1 | 0.218067321 | 0.206962728 | 0.972881356 |
| AW4EXXK | -1 | 0.407614929 | 0.380905892 | 0.948568089 |
| AWA2KJK | -2 | 0.504295305 | 0.469122208 | 0.960841613 |
| AWRBUQZ | -2 | 0.348714635 | 0.318765441 | 0.989246055 |
| AX3KEW9 | -2 | 0.508648683 | 0.466224953 | 0.972998247 |
| AX6281V | -1 | 0.484778598 | 0.451470151 | 0.976914085 |
| AXCO7I9 | -2 | 0.405069604 | 0.354378135 | 0.989129164 |
| AXD3W8O | -1 | 0.550576436 | 0.535545964 | 0.969900643 |
| AXDWDEA | -1 | 0.205399804 | 0.16905398 | 0.976446523 |
| AXI1PBS | -2 | 0.572993673 | 0.52706524 | 0.979135009 |
| AY8TPMP | -1 | 0.687388919 | 0.665070586 | 0.992752776 |
| AYEFCWQ | -1 | 0.338694614 | 0.310862289 | 0.969842198 |
| AYVQUF1 | -2 | 0.501009881 | 0.452443735 | 0.973173583 |
| AYWIEKR | -2 | 0.475534987 | 0.442181758 | 0.983752192 |
| AZ2RYW7 | -2 | 0.462640402 | 0.419668547 | 0.990005845 |
| AZ35PI5 | -2 | 0.190317585 | 0.167486191 | 0.983284629 |
| AZIK4ZA | -2 | 0.627991625 | 0.571335667 | 0.982057276 |
| AZKZ0AI | -1 | 0.558288749 | 0.523446065 | 0.992694331 |

### Supplementary Table 2. Parameters in LagCI analysis for demo datasets.

| program | time_tol (h) | step (h) | align_method | cor_method |
| --- | --- | --- | --- | --- |
| wearable_analysis | 0.5 | 1/60 | linear | spearman |
| omics_analysis | 12.25 | 30/60 | linear | spearman |

### Supplementary Table 3. Empirical runtime and memory scaling of lagCI.

| Analysis | Empirical exponent | R² | Interpretation |
| --- | --- | --- | --- |
| Runtime vs series length n | 0.50 | 0.97 | Slow growth over tested range |
| Runtime vs number of lags L | 0.41 | 0.90 | Runtime increases with lag window |
| Memory vs series length n | 0.90 | 0.99 | Approximately linear memory scaling |

### Supplementary Table 4. Top-k recovery in simulated datasets with known ground truth.

| Metric | Result |
| --- | --- |
| Precision@10 | 1.00 |
| Precision@20 | 1.00 |
| Precision@50 | 1.00 |
| Overall average precision (AP) | 0.86 |
| Direct AP | 1.00 |
| Feedback AP | 1.00 |
| Chain AP | 0.81 |
| Fork AP | 0.81 |
